# Hepatic Macrophages in Chronic Hepatitis B: Balancing Immunity and Pathology

**DOI:** 10.3390/biology15010076

**Published:** 2025-12-31

**Authors:** Anup S. Pathania, Sajad A. Bhat, Lukman A. Adepoju, Kusum K. Kharbanda, Natalia A. Osna

**Affiliations:** 1 Department of Pharmacology and Experimental Neuroscience, University of Nebraska Medical Center, Omaha, NE 68198, USA; 2 Research Service, Veterans Affairs Nebraska-Western Iowa Health Care System, Omaha, NE 68105, USA; 3 Department of Internal Medicine, University of Nebraska Medical Center, Omaha, NE 68198, USA

**Keywords:** chronic hepatitis B, HBV, macrophages, Kupffer cells, innate immunity, macrophage polarization, liver fibrosis, cytokines, TLR agonists, immune checkpoint inhibitors

## Abstract

Chronic HBV infection remains a global health challenge, driving liver fibrosis, cirrhosis, and hepatocellular carcinoma (HCC). Liver injury is primarily mediated by host immune responses rather than direct viral cytotoxicity. Macrophages, including Kupffer cells, play dual roles in antiviral defense and disease progression. HBV skews macrophages toward an M2-like, immunosuppressive phenotype, promoting viral persistence and fibrogenesis via cytokines such as Interleukin (IL)-10 and Transforming growth factor-beta (TGF-β). Therapeutic strategies targeting macrophage polarization, including Toll-like receptor (TLR) agonists, immune checkpoint inhibitors, and nanoparticle-based systems, are under investigation. Addressing macrophage heterogeneity and the immunosuppressive hepatic microenvironment using advanced models is essential. Modulating macrophages offers a promising avenue to control HBV, restore immune balance, and mitigate liver injury. This review highlights the central role of macrophages in chronic HBV infection and explores emerging therapeutic strategies.

## Simple Summary

Hepatitis B virus (HBV) is a DNA virus that infects liver cells (hepatocytes) and can cause both acute and chronic hepatitis. In chronic infection, HBV interacts not only with liver-resident macrophages (Kupffer cells) but also with blood-derived monocytes that infiltrate the liver. The virus alters their immune function, prompting them to shift toward an anti-inflammatory, M2-like state that suppresses antiviral responses. This shift allows HBV to persist and promotes liver inflammation, fibrosis, and disease progression. Understanding how HBV manipulates Kupffer cells and monocyte-derived macrophages (MDMs) is key to developing therapies that restore immune balance. Targeting macrophage polarization represents a promising strategy to reduce viral persistence and liver injury in chronic HBV.

## 1. Introduction

HBV is a hepatotropic DNA virus from the Hepadnaviridae family that primarily infects hepatocytes and can cause both acute and chronic liver diseases [1]. The World Health Organization (WHO) estimated that 296 million people had chronic HBV infection in 2022, and nearly 1.1 million deaths occurred that year due to complications such as cirrhosis and HCC. In the same year, approximately 1.2 million acute HBV infections occurred worldwide, suggesting that acute cases account for roughly 0.4% of the total burden relative to chronic prevalence. This disparity reflects the transient and often asymptomatic nature of acute infection, with most adults clearing the virus within six months.

Despite the availability of a highly effective prophylactic vaccine and antiviral therapies, HBV remains a major global health challenge, especially in sub-Saharan Africa and East Asia, where perinatal or early-life transmission is common [2]. As of 2022, only about 13% of individuals with hepatitis B were diagnosed, and merely 3% received treatment. This was far below the global targets of diagnosing 60% and treating 50% of infected individuals by 2025 [3]. These gaps underscore the urgent need to strengthen prevention, screening, and treatment efforts worldwide.

HBV spreads through exposure to infected blood or body fluids, with vertical (mother-to-child) transmission being the most common route in high-endemic areas [4]. About 90% of infants infected at birth develop chronic infection if not given timely prophylaxis [5]. Universal infant vaccination, particularly the administration of a birth dose along with hepatitis B immunoglobulin (HBIG), has significantly lowered transmission rates, though inconsistent vaccine coverage continues to sustain the epidemic [6,7]. Therefore, the WHO has set ambitious targets to eliminate viral hepatitis by 2030 by reducing new infections and hepatitis-related deaths by 90% [8]. Achieving these goals, however, depends on equitable access to screening, immunization, and treatment, especially in marginalized and resource-limited communities.

**Figure 1:**
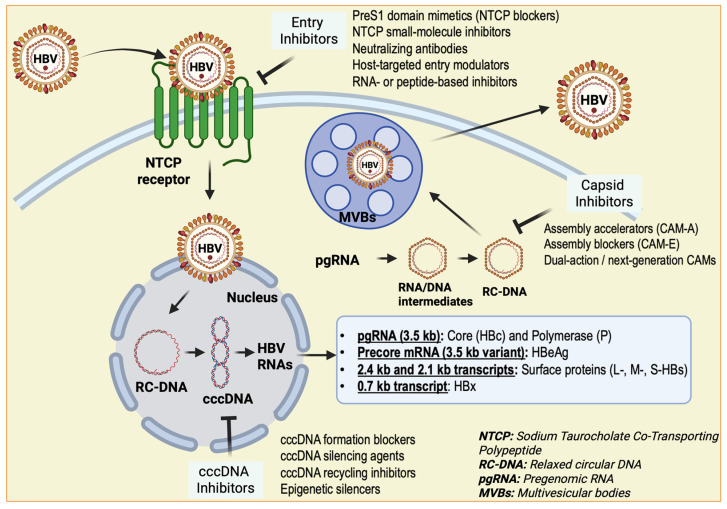
Replication cycle of HBV and potential intervention points. The replication process involves several key stages: initial binding and attachment of the virus to host cell receptors, penetration into the cell, biosynthesis of viral components, assembly of the nucleocapsid, and finally secretion of new viral particles [33,34]. Various treatment strategies target distinct steps of this cycle, including inhibitors of the endoplasmic reticulum that prevent capsid assembly [35]; cccDNA inhibitors that block the formation of the covalently closed circular DNA essential for viral transcription [36]; Epigenetic silencers (epidrugs) that suppress HBV transcription from cccDNA, thereby reducing viral RNA synthesis [37]; entry inhibitors that obstruct viral binding to host receptors such as NTCP and heparan sulfate proteoglycans (HSPG) [38]; and secretory protein inhibitors that hinder the release of HBV virions from infected cells [39].

HBV is a small, enveloped virus with a 3.2 kb partially double-stranded relaxed circular DNA (rcDNA) genome [9]. After entry into hepatocytes via the sodium taurocholate co-transporting polypeptide (NTCP) receptor, the viral genome is transported to the nucleus and converted into covalently closed circular DNA (cccDNA) [10]. This stable episomal form acts as a template for viral RNA transcription and persistence, contributing to chronic infection [11] (Figure 1). Chronic HBV infection, defined by the persistence of hepatitis B surface antigen (HBsAg) for more than six months, progresses through distinct phases marked by changes in viral replication, immune activity, and liver inflammation. The virus itself is minimally cytopathic; instead, liver injury results from an inadequate or dysregulated immune response to infected hepatocytes [12].

Immune control of HBV involves a coordinated interaction between innate and adaptive responses. In acute infection, strong activation of T and B cells helps clear the virus, while in chronic infection, immune exhaustion, tolerance, and expansion of regulatory T cells (Tregs) allow the virus to persist [13]. Among innate immune cells, macrophages, especially liver-resident Kupffer cells and infiltrating MDMs, play a key role in HBV pathogenesis [14,15,16]. They sense infection via pattern-recognition receptors (PRRs) such as TLR3, TLR4, and cytosolic DNA sensors, activating antiviral pathways including stimulator of interferon genes (STING)–TBK1–Interferon regulatory factor 3 (IRF3) and NOD-like receptor family pyrin domain-containing protein 3 (NLRP3) inflammasome signaling [17,18,19]. However, HBV can interfere with these antiviral defenses through viral proteins like Hepatitis B e antigen (HBeAg) and polymerase, which inhibit interferon signaling and promote immune evasion [20,21].

Mechanistically, TLR3/4 activation triggers early NF-κB and IRF3 signaling, inducing type I interferons and proinflammatory cytokines (TNF-α, IL-6) that prime NLRP3 inflammasome assembly through potassium efflux and mitochondrial reactive oxygen species (ROS) [22]. Cytosolic HBV DNA subsequently engages the cGAS-STING-TBK1-IRF3 axis, further amplifying IFN-β and interferon-stimulated gene expression [23,24,25,26]. Downstream, NLRP3 mediates maturation of IL-1β and IL-18; however, HBV subverts this cascade to promote persistence. HBeAg-driven metabolic reprogramming toward oxidative phosphorylation suppresses inflammasome activation, while HBeAg-TLR2-NF-κB signaling promotes a mixed inflammatory and profibrotic milieu [27,28]. In parallel, HBV polymerase binds STING and removes its K63-linked ubiquitination, thereby shutting down STING-dependent cytosolic DNA sensing and type I interferon induction [29]. Collectively, these pathways converge to impair IL-1β production despite intact initial sensing, thereby fostering immune tolerance during chronic infection.

This sustained impairment of macrophage antiviral function contributes directly to HBV progression, inflammation, and fibrosis. Changes in cytokine secretion, reduced phagocytosis, and impaired antigen presentation foster a microenvironment that supports viral persistence and progression to HCC [30,31,32]. Due to their plasticity, macrophages are promising targets for immunotherapy. Reprogramming macrophage activation or restoring antiviral signaling may improve immune clearance and supplement antiviral therapies aimed at suppressing viral replication and cccDNA maintenance.

## 2. Macrophages: Origin, Subtypes, and Functions

### 2.1. Monocyte-Derived vs. Tissue-Resident

Macrophages are highly versatile immune cells that play essential roles in host defense, tissue remodeling, and immune regulation. They originate from two primary sources: embryonic precursors and circulating monocytes. During embryogenesis, tissue-resident macrophages such as Kupffer cells in the liver, alveolar macrophages in the lungs, and microglia in the brain arise from yolk sac or fetal liver progenitors [40]. These cells populate tissues early in development and maintain themselves through local self-renewal, independent of bone marrow input.

In contrast, MDMs arise from hematopoietic stem cells in the bone marrow and are continuously replenished through the bloodstream [41]. Under conditions of infection, injury, or inflammation, circulating monocytes are recruited to affected tissues, where they differentiate into macrophages. In disease states, these infiltrating macrophages often complement or replace resident populations. For example, in chronic HBV infection, monocyte recruitment to the liver gives rise to inflammatory macrophages that contribute to hepatocellular injury and fibrosis [42].

### 2.2. Functional Polarization of Macrophages: M1 vs. M2 Phenotypes

A hallmark of macrophages is their ability to undergo functional polarization in response to environmental cues. Classically activated (M1) and alternatively activated (M2) macrophages represent two ends of a broad activation spectrum [43]. M1 macrophages are induced by stimuli such as tumor necrosis factor-alpha (TNF-α), lipopolysaccharide (LPS), or interferon-gamma (IFN-γ) and exhibit a pro-inflammatory phenotype. They produce high levels of cytokines, including IL-1β, IL-6, and TNF-α, as well as ROS and nitrogen (NO) species. This enables them to kill pathogens and tumor cells while sustaining inflammatory responses efficiently [44].

**Figure 2:**
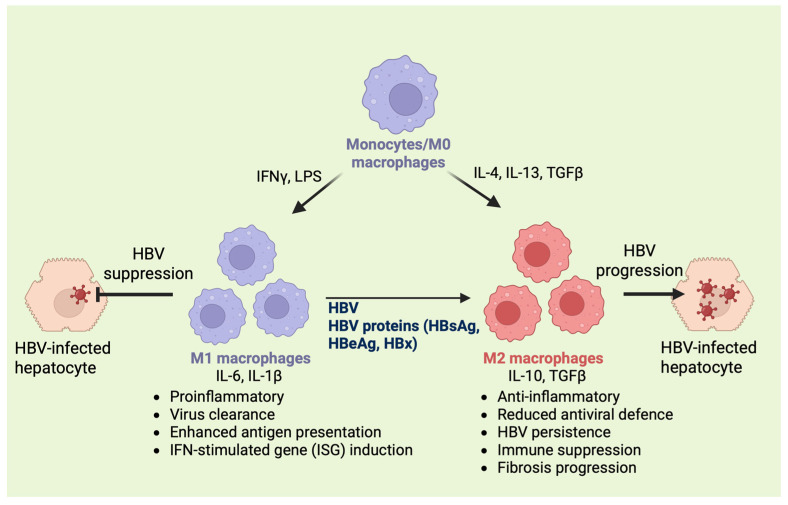
Schematic illustrates macrophage polarization and its role in HBV infection and disease progression. Circulating monocytes/M0 macrophages differentiate into classically activated M1 macrophages in response to IFNγ and LPS, or into activated M2 macrophages in response to IL-4, IL-13, and TGFβ. M1 macrophages produce proinflammatory cytokines (IL-6, IL-1β), promote antiviral responses, enhance antigen presentation, induce interferon-stimulated genes (ISGs), and contribute to HBV suppression in infected hepatocytes [103]. However, HBV and HBV-derived proteins (HBsAg, HBeAg, HBx) skew macrophage polarization toward the M2 phenotype. M2 macrophages secrete IL-10 and TGFβ, exhibit anti-inflammatory and immunosuppressive functions, and dampen antiviral defenses. This facilitates HBV persistence and replication, fibrosis progression, and disease advancement in HBV-infected hepatocytes [104,105,106].

In contrast, M2 macrophages are activated by cytokines such as IL-4, IL-10, and IL-13 and adopt an anti-inflammatory, tissue-repairing phenotype. They secrete IL-10, TGF-β, and other factors that promote extracellular matrix remodeling and fibrosis. While M2 macrophages are crucial for resolving inflammation, prolonged activation in chronic infections like HBV can promote immune suppression and fibrogenesis [45]. In HBV-infected livers, an imbalance between M1 and M2 macrophage populations can alter the hepatic microenvironment, driving persistent inflammation or pathological tissue remodeling (Figure 2). Understanding these dynamic polarization states is essential for unraveling HBV immunopathogenesis and identifying potential immunotherapeutic targets [15,46].

### 2.3. Role of Macrophages in HBV Immunity and Tissue Homeostasis

Hepatic macrophages, comprising liver-resident Kupffer cells and MDMs, play a central role in orchestrating anti-HBV immunity and maintaining liver homeostasis. Together, they form a dynamic network that balances antiviral defense, immune tolerance, and tissue repair. Depending on their activation state and the microenvironmental cues, hepatic macrophages can exhibit both antiviral and proviral functions. Early studies using the Duck Hepatitis B virus (DHBV) model demonstrated that non-parenchymal cell-derived cytokines, likely produced by hepatic macrophages, can inhibit viral replication in hepatocytes following endotoxin stimulation [47].

Supporting this antiviral role, macrophages secrete IL-6 upon HBV exposure and activate hepatocyte MAPK pathways that suppress the transcription factors hepatocyte nuclear factor (HNF1)α and HNF4α, essential for HBV replication [48]. Additionally, macrophage-derived TGF-β1 represses HNF4α expression and HBV core promoter activity, further inhibiting viral replication [49,50]. These findings underscore the capacity of hepatic macrophages to restrict HBV replication during the early phase of infection.

Conversely, macrophages can also adopt an immunoregulatory phenotype characterized by elevated IL-10 production, which suppresses antiviral T-cell responses and promotes HBV persistence. Experimental depletion of hepatic macrophages in mice before HBV exposure prevents the establishment of chronic infection. This is primarily due to the loss of IL-10-mediated suppression of the humoral immune response [51]. In addition, HBV has been shown to induce matrix metalloproteinase-9 (MMP-9) expression in peripheral blood mononuclear cells (PBMCs) and macrophages, leading to the inhibition of interferon type 1 receptor signaling (IFNAR1) and attenuation of interferon-mediated antiviral responses, thereby facilitating viral replication [52].

Kupffer cells, the largest population of tissue-resident macrophages in the body, serve as the first line of defense against HBV infection [53]. They express a broad array of PRRs, including TLRs, C-type lectins, and scavenger receptors, allowing them to detect pathogen-associated (PAMPs) and damage-associated molecular patterns (DAMPs) [54]. Upon recognizing HBV components, Kupffer cells secrete pro-inflammatory cytokines such as TNF-α, IL-6, and IL-1β, which help restrict viral replication and recruit additional immune cells to the liver [55]. They also function as antigen-presenting cells (APCs), expressing MHC class I and II molecules along with necessary costimulatory signals to activate T cells [56].

However, beyond initiating immune responses, Kupffer cells play an essential role in maintaining hepatic immune tolerance. They suppress cytotoxic CD8^+^ T cell activity, promote the expansion of Tregs, and efficiently clear apoptotic cells and gut-derived microbial products without provoking excessive inflammation [57,58]. Following phagocytosis, Kupffer cells release immunosuppressive mediators such as TGF-β, IL-10, and prostaglandin E2, creating a localized immunosuppressive environment that limits lymphocyte activation and preserves liver homeostasis [59]. During chronic HBV infection, Kupffer cells acquire a tolerogenic phenotype marked by elevated IL-10 and TGF-β secretion and decreased expression of costimulatory molecules. This phenotypic shift fosters viral persistence, dampens antiviral T cell activity, and promotes ongoing inflammation, fibrosis, and hepatocarcinogenesis [51,60,61].

Furthermore, Kupffer cells can be categorized into distinct functional subsets based on the expression of the macrophage receptor with collagenous structure (MARCO): MARCO^+^ and MARCO^−^ [62,63]. MARCO^+^ Kupffer cells are largely resident, tolerogenic macrophages that contribute to pathogen clearance and the maintenance of liver homeostasis. They express high levels of pro-repair markers such as Gpnmb and Mertk, and regulate proinflammatory signaling via scavenger receptor activity, thereby promoting anti-inflammatory and tissue-resolving responses [64]. In contrast, MARCO^−^ Kupffer cells are typically more inflammatory and often include MDMs that expand during liver injury or infection. These cells exhibit lower expression of repair mediators and are associated with acute injury responses or M2-like polarization under pathological conditions, including hepatocellular carcinoma [63,64]. Thus, MARCO serves as a critical marker distinguishing pro-resolution (MARCO^+^) from potentially pathogenic (MARCO^−^) Kupffer cell subsets, with MARCO deficiency impairing repair macrophage accumulation and exacerbating liver damage.

In the context of HBV infection, direct studies comparing MARCO^+^ and MARCO^−^ Kupffer cells remain limited, but current evidence highlights the general role of Kupffer cells in viral persistence. MARCO^+^ Kupffer cells, as tolerogenic subsets, likely support HBV persistence by clearing apoptotic hepatocytes through scavenger receptor activity, secreting IL-10 to suppress antiviral CD8^+^ T cell responses and to limit excessive inflammation that could otherwise lead to viral clearance. Conversely, MARCO^−^ Kupffer cells may transiently drive pro-inflammatory responses via cytokines such as TNF-α and CXCL10, which can potentially aid early viral control. However, they lack the tolerance-inducing capacity of MARCO^+^ cells and may contribute to liver pathology if these inflammatory responses are unchecked. Collectively, this inferred dichotomy suggests that MARCO^+^ dominance favors chronic immune tolerance, while MARCO^−^ subsets skew toward inflammatory responses and tissue injury or resolution.

Overall, Kupffer cells exhibit a dual role during HBV infection by initiating antiviral and immune-activating responses during acute infection, while driving immune regulation, tolerance, and tissue remodeling in chronic disease.

In contrast, MDMs are recruited from circulating monocytes in response to inflammation and infection, primarily through the C-C chemokine receptor 2 (CCR2)/monocyte chemoattractant protein-1 (CCL2) axis [65]. Once in the liver, they tend to adopt a more inflammatory phenotype than resident macrophages. They produce mediators such as IL-18, TGF-β, and galectin-3, exacerbating hepatocellular injury and promoting fibrogenesis via activation of hepatic stellate cells (HSC) through platelet-derived growth factor (PDGF) and TGF-β signaling [66,67]. Notably, even after antiviral therapy suppresses HBV replication, these inflammatory MDMs often persist, preserving a proinflammatory milieu that sustains tissue injury.

However, MDMs also display functional plasticity and a dual role in chronic HBV pathogenesis. While proinflammatory subsets can drive liver damage and fibrosis, other subsets can shift toward reparative or regulatory phenotypes that facilitate tissue remodeling, resolution of inflammation, and fibrosis regression by secreting matrix metalloproteinases and anti-inflammatory mediators [15,66]. This context-dependent plasticity is increasingly appreciated as central to macrophage biology in chronic liver disease. Experimental models further show that M1-like macrophages exhibit stronger HBV suppression, whereas M2-like or immunosuppressive macrophages tend toward tolerance and persistence of viral infection [31,68].

Single-cell and transcriptomic analyses reveal that distinct macrophage subpopulations exhibit both fibrogenic and pro-resolving signatures during fibrosis progression and regression, and HBV-induced metabolic reprogramming can influence macrophage polarization state [66,69]. HBV suppresses glycolysis in macrophages, including M1-like subsets, in contrast to the high-glycolysis profile observed in infections such as Hepatitis C virus (HCV) [27]. In mouse Kupffer cells and human models, wild-type HBV increases oxygen consumption (OCR) via HBeAg, whereas HBeAg-null mutants enhance glycolysis [27]. This high OXPHOS/low glycolysis state in HBV-exposed M1-like macrophages reduces IL-1β production, which would otherwise limit HBV by downregulating hepatocyte PPARα and FOXO3, thereby promoting viral persistence [68]. Lipid metabolites further drive phenotypic changes in portal areas, bile ducts, and lipid-rich zones, including expansion of pro-inflammatory Trem2^+^ macrophages in chronic HBV-associated acute-on-chronic liver failure (CHB-ACLF) [70,71]. Together, these glucose and lipid metabolic adaptations foster mixed M1/M2 macrophage features, impair viral clearance, and exacerbate liver inflammation.

Collectively, hepatic macrophages act as a double-edged sword during HBV infection, mediating viral clearance while also contributing to immune-mediated tissue injury and fibrosis. Understanding the molecular and environmental cues that regulate macrophage activation and polarization will be critical for developing therapeutic strategies to restore immune balance. Targeting macrophage metabolism, immune checkpoints, or reprogramming their phenotype toward a reparative state holds promise for mitigating HBV-associated liver disease.

## 3. Immune Evasion Mechanisms Used by HBV to Subvert Macrophage Functions

HBV establishes persistent infection primarily by evading or suppressing host immune responses, including those orchestrated by macrophages. Several HBV proteins directly interfere with macrophage signaling pathways [72]. Most notably, the viral polymerase and HBx inhibit interferon production by disrupting the activation of interferon regulatory factors IRF3 and IRF7, which are essential for initiating antiviral cytokine responses. HBx also impairs mitochondrial antiviral signaling (MAVS) and interferes with retinoic acid-inducible gene-I (RIG-I)-like receptor pathways, further dampening antiviral signaling [73]. In addition to these intracellular disruptions, HBV employs immune-evasion strategies through surface-antigen-mediated interactions. HBsAg has been shown to inhibit TLR4 and TLR2 signaling by blocking downstream molecules in their respective pathways rather than by reducing TLR expression. It disrupts TLR4 signaling through upregulation of A20, a negative regulator of TLR signaling, and also selectively inhibits TLR2 ligand-induced IL-12 production by interfering with JNK activation [74,75].

Moreover, HBV upregulates programmed death ligand 1 (PD-L1) expression on macrophages, which binds PD-1 expressed on T cells. This interaction drives T cell exhaustion, enabling viral persistence during chronic infection. This immune checkpoint engagement not only prevents cytotoxic T cell-mediated elimination of infected hepatocytes but also fosters an immunosuppressive hepatic microenvironment [76,77]. This environment is further marked by the expression of HepPar-1 (Hepatocyte Paraffin 1), a diagnostic marker associated with HCC.

In addition to modulating immune checkpoints, HBV reprograms the metabolic landscape of macrophages to suppress their pro-inflammatory activity. The virus induces hyperacetylation of key metabolic enzymes, including citrate synthase and pyruvate dehydrogenase. This disrupts the tricarboxylic acid (TCA) cycle and drives macrophages toward an anti-inflammatory (M2-like) phenotype that promotes immune tolerance and viral persistence [78]. By hijacking these metabolic pathways, HBV alters macrophage function, contributing to both a pro-inflammatory environment and sustained viral persistence and liver pathology. Specialized hepatic macrophage subsets, such as Trem2^+^ cells, display altered activation in HBV-infected livers, further modulating the balance between tissue injury and repair [31].

Recent studies have also shown that HBV exploits small extracellular vesicles (sEVs) released from infected hepatocytes to further impair macrophage function. These HBV-enriched sEVs interact with M1-polarized macrophages and suppress critical pro-inflammatory mediators, including TLR4, NLRP3, pro-caspase-1, active caspase-1 (p20), IL-1β, and IL-18; thereby attenuating innate immune activation [79,80]. This immunosuppressive effect is partly mediated by elevated levels of miR-146a and flap endonuclease-1 (FEN-1) contained within HBV-associated vesicles, which inhibit inflammatory signaling and facilitate viral persistence [79]. Furthermore, these vesicles deliver immunoregulatory microRNAs that downregulate IL-12p35 mRNA expression in macrophages, weakening innate immune responses and impairing antiviral cytokine production [81].

Collectively, these mechanisms illustrate how HBV co-opts macrophage signaling, metabolic programming, and extracellular vesicle communication to evade immune detection and sustain chronic infection. Moreover, these multifaceted immune evasion strategies not only allow HBV to persist within the host but also set the stage for chronic liver injury. This highlights the central role of macrophages in mediating HBV-induced hepatic damage, as discussed in the following section.

## 4. Macrophages and HBV-Induced Liver Damage

In chronic HBV infection, macrophages remain persistently activated due to ongoing hepatocyte damage and sustained exposure to viral antigens. This chronic activation drives hepatic inflammation through the recruitment of MDMs and the activation of resident Kupffer cells [82]. Activated macrophages perpetuate inflammatory signaling by secreting chemokines such as CCL2, which attract additional immune cells to the liver. Prolonged inflammation shifts macrophage function from immune surveillance toward tissue remodeling and fibrosis promotion [83]. Direct cell-to-cell contact and paracrine signaling between macrophages and HSCs, the primary fibrogenic cells in the liver, stimulate HSCs to release pro-fibrotic factors, particularly TGF-β. TGF-β activates HSCs to secrete collagen, an extracellular matrix protein, resulting in excessive matrix deposition, fibrotic scarring, and disruption of normal liver architecture, ultimately progressing toward liver fibrosis [84].

### 4.1. Role of Macrophage-Derived Cytokines in Liver Damage

Macrophages influence liver pathology through a finely tuned but often disrupted cytokine network. The cytokine profile of macrophages comprises a diverse range of signaling molecules that intricately regulate immune responses, inflammation, and tissue homeostasis. Key pro-inflammatory cytokines produced include TNF-α and IL-6, which play essential roles in initiating and sustaining inflammatory reactions, recruiting immune cells, and activating other components of the immune system. IL-6 additionally has pleiotropic effects, influencing both immune activation and tissue repair processes. Balancing these pro-inflammatory signals are anti-inflammatory cytokines such as IL-10 and TGF-β. IL-10 serves as a major immunoregulatory cytokine that limits excessive inflammation by inhibiting the synthesis of pro-inflammatory cytokines and promoting immune tolerance. TGF-β contributes to the resolution of inflammation by suppressing immune cell activation and supporting tissue remodeling and repair.

**TNF-α:** TNF-α, predominantly produced by M1 macrophages, orchestrates inflammatory responses and promotes innate immune activity against HBV. It recruits and activates T cells, neutrophils, and natural killer (NK) cells by inducing adhesion molecules and chemokines. However, sustained TNF-α signaling contributes to hepatocellular injury by activating TNFR1-mediated apoptosis [85]. While this mechanism aids viral clearance, persistent TNF-α activity damages the liver parenchyma, promotes fibrosis, increases oxidative stress, and upregulates matrix metalloproteinases (MMPs). Therapeutic strategies that fine-tune TNF-α activity, rather than globally suppress it, may preserve antiviral effects while minimizing tissue damage [86].

**IL-6:** IL-6, abundantly secreted by M1 macrophages and Kupffer cells, plays multiple roles, including activating the acute phase response, promoting T and B cell differentiation, and supporting hepatocyte survival and proliferation [87,88]. In acute infections, IL-6 contributes to viral clearance by enhancing immune activation. In chronic HBV, prolonged IL-6 elevation drives immune dysregulation, including impaired antiviral T cell responses and expansion of regulatory T cells [89]. IL-6 also promotes hepatocyte proliferation, angiogenesis, and resistance to apoptosis via STAT3 signaling. This creates a pro-tumorigenic and fibrogenic environment that can facilitate HCC [90].

**IL-10:** IL-10, primarily produced by M2 macrophages, regulatory T cells, and dendritic cells, is a potent anti-inflammatory cytokine that limits excessive immune activation [91]. It inhibits the synthesis of pro-inflammatory cytokines (e.g., TNF-α, IL-1β), downregulates antigen presentation, and suppresses overactivation of T cells and macrophages. In chronic HBV, elevated IL-10 contributes to immune tolerance, viral persistence, and a microenvironment prone to chronic inflammation and fibrosis. Therapeutic modulation of IL-10 requires careful balancing to enhance antiviral immunity without triggering autoimmune-like liver injury [92].

**TGF-β:** M2 macrophages and activated HSCs secrete TGF-β, a central mediator of liver fibrogenesis. TGF-β promotes HSC activation, proliferation, and their transformation into myofibroblast-like cells, leading to excessive collagen deposition and architectural distortion of the liver. Beyond fibrosis, TGF-β exerts immunosuppressive effects by inhibiting T cell proliferation, reducing MHC class II expression, and promoting regulatory T cell expansion [93]. Persistent TGF-β signaling during chronic HBV infection strongly associates with cirrhosis and HCC, making it a key therapeutic target. However, clinical modulation of TGF-β must be approached cautiously to maintain tissue homeostasis and immune balance, avoiding unintended adverse effects [94].

Studies have shown that liver-resident macrophages exposed to HBV antigens preferentially produce TGF-β, promoting an anti-inflammatory milieu. Additionally, TGF-β facilitates the expansion of Tregs that suppress antiviral immune responses, contributing to viral persistence and chronic liver damage [93]. The role of TGF-β in activating HSCs and promoting fibrosis is well documented, underscoring its pivotal role in disease progression from chronic inflammation to fibrosis and ultimately to HCC. Therefore, targeting TGF-β signaling is promising but requires careful balancing to preserve normal immune function and prevent progression of fibrosis.

### 4.2. Cytokine Balance: A Determinant of Disease Outcome

The amount, timing, and balance of macrophage-derived TNF-α, IL-6, IL-10, and TGF-β are critical determinants of liver pathology in chronic HBV infection. Early and appropriately regulated cytokine production can promote viral clearance and immune resolution. In contrast, chronic or skewed cytokine release leads to persistent inflammation, immune cell exhaustion, fibrosis, and hepatocarcinogenesis. In chronic HBV, the cytokine milieu often reflects a maladaptive immune response, characterized by excessive immunosuppression mediated by IL-10 and TGF-β, alongside unresolved inflammation driven by TNF-α and IL-6. This imbalance fosters a hepatic microenvironment that supports viral persistence and progressive liver injury [95,96,97]. Therapeutic strategies aimed at modulating macrophage cytokines represent a rational approach to restoring immune competence and preventing the progression of liver disease. Future interventions should focus on reestablishing cytokine balance, achieving antiviral efficacy while preserving liver regeneration, and minimizing immunopathology.

### 4.3. Role of Macrophages in Liver Repair

Although HBV is a non-cytopathic virus, liver injury in chronic infection arises primarily from immune-mediated mechanisms. Beyond cytokine signaling, activated macrophages directly contribute to hepatocyte damage by releasing ROS and NO, which trigger oxidative stress, mitochondrial dysfunction, and apoptotic cell death [98,99]. These cytotoxic effects amplify hepatocellular injury, perpetuate inflammation, and further compromise liver integrity.

Paradoxically, macrophages also play an essential role in liver tissue repair. They clear apoptotic cells through efferocytosis and secrete anti-inflammatory cytokines and growth factors that promote hepatocyte regeneration [100]. However, in chronic HBV infection, the persistent inflammatory and fibrotic milieu impairs effective regeneration. The reparative process becomes dysfunctional or dysregulated, tilting the balance toward progressive liver damage rather than recovery [101,102].

Overall, the remarkable plasticity of hepatic macrophages underscores their dual role in HBV-induced liver injury. They act as both mediators of hepatocellular injury and key drivers of liver repair. Therapeutic approaches that fine-tune macrophage activation and cytokine profiles hold promise for restoring immune balance, enhancing regeneration, and mitigating disease progression.

## 5. Single-Cell Transcriptomic Studies Identifying Distinct Macrophage Subsets in HBV or HBV-Related HCC

Single-cell RNA sequencing (scRNA-seq) of PBMCs from five CHB patients with liver fibrosis and three healthy controls revealed a marked increase in the overall proportion of monocytes in CHB patients compared with healthy individuals. Within the monocyte compartment, classical, intermediate, and non-classical subsets were all expanded in CHB. However, there was a relative decrease in classical monocytes and an increase in non-classical monocytes, suggesting a shift toward patrolling, inflammation-associated phenotypes. A distinct monocyte subset characterized by monocyte-platelet aggregates (MPAs) was identified and was significantly enriched in CHB patients with liver fibrosis compared with healthy controls. MPA frequency was higher in CHB-associated liver injury than in non-HBV liver fibrosis or HBV-related liver cancer, indicating a specific association with CHB-driven fibrotic inflammation [107].

Another study integrating scRNA-seq with bulk RNA-seq from liver tissues of cirrhosis and HCC patients identified macrophage transcriptomic changes associated with progression to HCC. scRNA-seq revealed distinct macrophage subtypes and dynamic shifts in gene expression. Eleven macrophage-related genes showed significant expression changes. KLK11, MARCO, CFP, KRT19, GAS1, SOD3, and CYP2C8 were downregulated, consistent with loss of tumor-suppressive and pro-apoptotic functions. In contrast, TOP2A, CENPF, MKI67, and NUPR1 were upregulated, indicating increased proliferative activity and a shift toward M2 macrophage polarization. These transcriptional changes suggest macrophage remodeling that fuels cirrhosis-to-HCC progression [108].

ScRNA-seq analyses of HBV-associated HCC further reveal a profoundly immunosuppressive tumor microenvironment. Exhausted CD8^+^ T cells dominate and express high levels of PD-1, TIM-3, and LAG-3. Tumor-associated macrophages (TAMs) are skewed toward an M2 phenotype, secrete IL-10 and TGF-β, and upregulate arginase-1, leading to arginine depletion and suppression of T cell function. Tregs further inhibit NK cell and CD8^+^ T cell activity. Malignant hepatocytes exhibit marked heterogeneity, including HBV integration-driven proliferative populations activated through Wnt/β-catenin signaling and stem-like, therapy-resistant cells. Peri-tumoral regions are enriched in antiviral immune effectors, whereas tumor cores are largely immune-depleted. Crosstalk between TAMs and tumor cells, particularly through the CCL2-CCR2 axis, promotes tumor progression and metastasis. Notably, TIGIT-NECTIN2 interactions between T cells and tumor cells or antigen-presenting cells are more prominent than PD-1 signaling. In mouse models, TIGIT blockade reduces tumor growth, increases CD4^+^ and CD8^+^ T cell infiltration, and reverses T cell exhaustion. This highlights TIGIT inhibition, alongside PD-1 blockade and TAM repolarization, as promising immunotherapeutic strategies for HBV-associated HCC [109].

Another study integrating Viral-Track, a tool for detecting viral integrations, with scRNA-seq profiling of HBV-associated HCC tumors uncovered unexpected HBV lymphotropism, with infection detected in immune cells such as B and T cells in addition to hepatocytes. HBV-HCC tumors exhibited depletion of NK cells, macrophages, and dendritic cells, alongside enrichment of malignant hepatocytes, compared with non-HBV-HCC tumors. TAMs in HBV-associated HCC overexpressed metallothioneins, particularly MT1G, and this expression was associated with poor prognosis. HBV-infiltrated CD8^+^ T cells exhibited impaired cytotoxic function, marked by increased MDK and CTLA4 expression, reduced IFN-γ production, and a predominance of immunosuppressive ligand-receptor interactions compared with antitumor interactions in non-HBV-HCC [110].

Collectively, these findings highlight HBV-induced immune evasion, lymphotropism, and the mechanisms sustaining viral persistence in HCC. These single-cell analyses uncover extensive immune remodeling in CHB and HBV-related HCC, with macrophages emerging as key drivers of disease progression. This immune reprogramming fosters viral persistence, tumor growth, and immune escape, underscoring macrophage modulation and immune checkpoint inhibition as promising therapeutic avenues for HBV-associated HCC.

## 6. Therapeutic Implications

Although current antiviral therapies have markedly reduced HBV replication and slowed disease progression, achieving a complete cure remains challenging. The persistence of cccDNA within hepatocytes serves as a stable viral reservoir that nucleos(t)ide analogues (NAs) cannot eliminate. This latent pool enables viral rebound once therapy is withdrawn and hinders complete immune reconstitution despite long-term viral suppression. Possible treatment targets for antivirals are summarized in Figure 1. Moreover, immune dysfunction is characterized by exhausted T cell responses, and persistent immunosuppressive cytokine signaling further impedes the clearance of infected hepatocytes.

Given these limitations, therapeutic strategies are increasingly shifting toward host-directed approaches. Targeting macrophages represents a promising avenue, as they are key regulators of immunity in chronic HBV infection. Modulating macrophage recruitment, activation, and polarization may help restore antiviral immunity, reduce fibrosis, and improve treatment outcomes. Efforts to integrate macrophage-targeted interventions with existing antiviral regimens hold potential to overcome current therapeutic barriers and move closer to achieving a functional cure (Table 1).

### 6.1. TLR Agonists

TLRs are crucial pattern recognition receptors expressed on macrophages that detect viral components and initiate innate immune responses. Agonists of TLR7 and TLR8, such as GS-9620 (vesatolimod) and selgantolimod, are currently being explored for their potential to stimulate macrophages and other innate immune cells. Activation of these pathways induces the production of type I interferons and pro-inflammatory cytokines, thereby enhancing antiviral immunity. Preclinical HBV models demonstrate that TLR agonists can suppress viral replication, promote cccDNA degradation, and restore immune responsiveness by reversing T cell and macrophage exhaustion [111]. By reinvigorating innate immune activation, these agents may help overcome immune tolerance and limit viral persistence.

Ongoing clinical trials are assessing their safety, tolerability, and efficacy as adjunctive therapies to existing antiviral regimens [112]. However, some completed trials show that, despite immune activation, therapies such as GS-9620, when combined with nucleos(t)ide analogs, fail to achieve meaningful HBsAg reduction, limiting functional cure. Systemic dosing causes off-target cytokine-mediated toxicities, including flu-like symptoms, elevated liver enzymes, and cytopenias, while HBV-driven TLR downregulation and transient antiviral responses further compromise durable efficacy [113,114].

### 6.2. CCR2/CCL2 Inhibitors

During chronic liver inflammation, CCL2 recruits CCR2^+^ inflammatory monocytes from the bone marrow to the liver, where they differentiate into pro-inflammatory and pro-fibrotic macrophages [115]. Blocking the CCR2-CCL2 signaling axis, for example, with cenicriviroc, reduces macrophage infiltration, dampens hepatic inflammation, and limits fibrosis progression. By preventing the accumulation of MDMs while preserving Kupffer cell homeostasis, these inhibitors may attenuate liver injury without compromising essential immune defense. Cenicriviroc has shown anti-fibrotic activity in non-alcoholic steatohepatitis (NASH) clinical studies, and its therapeutic potential in HBV-induced fibrosis is currently under investigation [116,117].

### 6.3. Immune Modulators Targeting Macrophage Function

Immune modulators that enhance or reprogram macrophage activity play a crucial role in strengthening anti-HBV immunity. By modulating macrophage phenotypes and functions, these agents can shift the immune response from a tolerant or immunosuppressive state toward one capable of effective viral control. Some examples are:

**IFN-α:** Pegylated IFN-α remains among the earliest immunotherapeutic approaches for HBV management. Beyond its antiviral effects on hepatocytes, IFN-α profoundly influences macrophage activation. It stimulates macrophages, dendritic cells, and NK cells to produce type I interferons and pro-inflammatory cytokines, enhancing innate antiviral defenses. In macrophages, IFN-α promotes M1 polarization, boosting phagocytic and antigen-presenting functions critical for antiviral immunity. However, the clinical use of IFN-α is constrained by systemic toxicity, limited efficacy in immunotolerant patients, and variable responsiveness due to the immunosuppressive hepatic microenvironment [118].

**Immune checkpoint inhibitors:** In chronic HBV infection, macrophages frequently express PD-L1 that binds PD-1 on T cells and promotes T cell exhaustion and viral persistence [119]. Inhibiting the PD-1/PD-L1 axis using immune checkpoint inhibitors can not only restore antiviral T cell function but also reprogram macrophage behavior. Blocking PD-L1 reduces suppressive cytokine feedback (e.g., IL-10, TGF-β) and may partially reverse macrophage polarization toward an M1-like antiviral phenotype. However, given the delicate balance between immune clearance and tissue damage in the liver, such interventions must be applied with caution. Ongoing clinical trials are assessing the safety, efficacy, and hepatotoxicity of checkpoint blockade in chronic HBV infection [120,121].

**Therapeutic vaccines:** Therapeutic HBV vaccines aim to stimulate robust virus-specific T cell responses and reinvigorate antigen presentation, in which macrophages play a vital role. Acting as APCs, macrophages can process HBV antigens and promote adaptive immune activation. Advanced vaccine designs now incorporate adjuvants that activate TLR pathways or skew macrophages toward an M1 phenotype, enhancing antiviral cytokine production and immune activation. Additionally, macrophage activation through therapeutic vaccination may promote clearance of infected hepatocytes and reduction in viral reservoirs [122].

### 6.4. Nanoparticle-Based Strategies for Targeting Macrophages

Nanotechnology has revolutionized therapeutic delivery by enabling precise targeting of hepatic macrophages while minimizing systemic toxicity [123]. Because of their intrinsic phagocytic and endocytic capacity, macrophages represent ideal cellular targets for nanoparticle-mediated drug delivery in chronic HBV infection. One example is liposome-encapsulated therapeutics that have emerged as a powerful platform for macrophage-specific drug delivery. These vesicles can carry small molecules, peptides, or nucleic acids directly into macrophages. For instance, mannose-modified liposomes exploit the macrophage mannose receptor (CD206) to achieve selective uptake by Kupffer cells. Such systems have been used to deliver TLR agonists, siRNAs, and genome-editing agents that modulate macrophage activation and cytokine production [124]. By fine-tuning macrophage polarization, these liposomes can suppress chronic inflammation and promote antiviral immunity in HBV-infected livers [123].

Another example includes RNA-based nanocarriers, such as siRNA-loaded liposomes modified with PreS/2-21 peptides, which primarily target hepatocytes through the NTCP receptor. They also indirectly reshape macrophage-mediated immune responses by reducing viral replication and dampening liver inflammation in preclinical HBV models. These multifunctional nanoparticles simultaneously inhibit viral entry and replication while modulating the hepatic immune microenvironment, including macrophage activation dynamics [125].

Furthermore, recent hybrid nanoparticle platforms, combining lipid and polymeric materials, have been engineered to release drugs in response to inflammatory cues characteristic of chronically HBV-infected livers [126]. Such systems can reprogram macrophages from pro-fibrotic (M2-like) to restorative (M1-like or regulatory) phenotypes, thereby attenuating fibrosis and promoting liver repair. Additionally, size-controlled nanoparticles (<200 nm) facilitate passive targeting to the liver via macrophage uptake within the sinusoidal network. This property enhances intrahepatic drug accumulation and allows for efficient delivery of antiviral, antifibrotic, or immunomodulatory agents that act directly within macrophages [125]. These agents can also modulate macrophage-hepatocyte interactions, key processes in HBV-associated liver disease. Overall, macrophage-targeted nanotechnologies offer a promising approach to enhance antiviral immunity, reduce inflammation, and promote liver repair with minimal systemic toxicity.

**Table 1:** Therapeutic Strategies Targeting Macrophages in Chronic HBV Infection.

Therapeutic Strategy	Example Agents	Mechanism of Action	Therapeutic Effects
**Agents currently in clinical use (Approved/Standard of Care):**			
**Nucleos(t)ide Analogues**	Tenofovir, Entecavir	Inhibit HBV polymerase and reduce viral replication	Decrease viral load, prevent progression of liver disease [127,128]
**Interferon-Alpha (IFN-α)**	Pegylated IFN-α	Stimulate macrophages, NK cells, and dendritic cells to produce antiviral cytokines.	Induce viral suppression and immune restoration [129]
**Agents in clinical development:**			
**TLR Agonists**	GS-9620 (TLR7), Selgantolimod (TLR8)	Activate macrophages and innate immunity through type I interferon and pro-inflammatory cytokines.	Suppress HBV replication, enhance antiviral immune response [130,131]
**CCR2/CCL2 Inhibitors**	Cenicriviroc	Inhibit monocyte recruitment to the liver, reducing infiltration of pro-fibrotic macrophages.	Limit inflammation and liver fibrosis [132]
**CSF-1/CSF-1R Inhibitors**	Pexidartinib	Modulate or deplete macrophage subsets via CSF-1R inhibition	Suppress immunosuppressive macrophages and support immune activation [133]
**Immune Checkpoint Inhibitors**	Anti-PD-1, Anti-PD-L1	Restore exhausted T cells and downregulate PD-L1 expression on macrophages.	Enhance T cell activity, reduce immune suppression [134].
**Therapeutic Vaccines**	GS-4774, NASVAC	Stimulate virus-specific T cell responses and macrophage activation	Boost adaptive immunity and reduce HBV persistence [135]
**Preclinical/early translational approaches:**			
**Liposome-Encapsulated Agents**	Mannose-functionalized liposomes	Target mannose receptors on macrophages for selective uptake	Deliver siRNAs or immune modulators to hepatic macrophages [136]
**Ligand-Conjugated Nanoparticles**	CD163- or scavenger receptor-targeted systems	Enhance macrophage-specific delivery and reduce systemic toxicity in cancer models. Although CD163-targeted nanoparticles have not yet been tested in HBV, the upregulation of CD163 in hepatic macrophages during chronic HBV makes it a promising target for selective macrophage-specific delivery.	Improve therapeutic specificity and effectiveness [137].
**RNA-Based Therapeutics**	siRNA/mRNA nanoparticles	Silence fibrotic or immunosuppressive genes in macrophages	Reprogram macrophages and aid viral clearance [138]
**Hybrid/Stimuli-Responsive Systems**	Enzyme/pH-sensitive nanocarriers	Controlled release triggered by the inflammatory liver environment	Precision delivery to modulate macrophage activity in HBV livers [139]

## 7. Conclusions

Chronic HBV infection remains a significant global health issue, with liver cirrhosis and HCC as primary outcomes. Hepatic macrophages, including Kupffer cells and monocyte-derived cells, are vital in viral detection, immune regulation, and tissue repair. In chronic infection, macrophage dysfunction, characterized by a shift toward an M2-like phenotype, promotes immune tolerance, fibrosis, and disease progression (Figure 2). Therapeutically, restoring macrophage function through repolarization or targeted delivery systems offers a promising avenue. Strategies that aim to reprogram M2-like macrophages into antiviral M1 phenotypes or utilize macrophage-targeted nanoparticles could restore immune control and reduce fibrosis. Combining these macrophage-focused approaches with antiviral therapies may rebalance liver immunity, promote viral clearance, and support the development of curative treatments for chronic HBV infection.

## Data Availability

No new data were created or analyzed in this study.

## Abbreviations

cccDNA	Covalently closed circular DNA
HSC	Hepatic stellate cell
HBV	Hepatitis B virus
HCC	Hepatocellular carcinoma
HCV	Hepatitis C virus
HbcAg	Hepatitis B core antigen
HbsAg	Hepatitis B surface antigen
HSPG	Heparan sulfate proteoglycans
HBeAg	Hepatitis B e antigen
IRF3	Interferon regulatory factor 3
ISG	Interferon-stimulated genes
IL-18	Interleukin-18
IL-1β	Interleukin-1 beta
IL-6	Interleukin-6
EVs	Extracellular vesicles
MDMs	Monocyte-derived macrophages
NTCP	Sodium taurocholate co-transporting polypeptide
NLRP3	NOD-like receptor family pyrin domain-containing protein 3
NASH	Non-alcoholic steatohepatitis
rcDNA	Relaxed circular DNA
CX3CL1	C-X3-C motif chemokine ligand 1
CX3CR1	C-X3-C motif chemokine receptor 1
CCL2	Monocyte chemoattractant protein-1
DAMPs	Damage-associated molecular patterns
TLR	Toll-like receptor
TCA	Tricarboxylic acid cycle
PRRs	Pattern recognition receptors
PAMP	Pathogen-associated molecular patterns
PDGF	Platelet-derived growth factor
PD-L1	Programmed death ligand 1
TNF-α	Tumor necrosis factor-alpha
TGF-β	Transforming growth factor-beta
ROS	Reactive oxygen species
STING	Stimulator of interferon genes
RIG-I	Retinoic acid-inducible gene I
CSF-1	Colony-stimulating factor 1
WHO	World Health Organization
TAMs	Tumor-associated macrophages
